# Trajectories of Loneliness Among Older Women and Men: Variation by Sexual Identity?

**DOI:** 10.1093/geront/gnac058

**Published:** 2022-04-22

**Authors:** Jack Lam, Alice Campbell

**Affiliations:** Institute for Social Science Research, University of Queensland, Brisbane, Queensland, Australia; Australian Research Council of Excellence on Children and Families over the Life Course, Brisbane, Queensland, Australia; Institute for Social Science Research, University of Queensland, Brisbane, Queensland, Australia; Australian Research Council of Excellence on Children and Families over the Life Course, Brisbane, Queensland, Australia

**Keywords:** Australia, Growth models, Resilience, Social disadvantage

## Abstract

**Background and Objectives:**

The aim of this study is to contribute to the literature on variation in later-life outcomes by sexual identity. Drawing on the Iridescent Life Course framework, we examined differences in loneliness trajectories, and tested the roles of social connectedness and support, and socioeconomic and health statuses in explaining any observed disparities.

**Research Design and Methods:**

Using growth models, we analyzed 19 years of data (2001–2019) from adults aged 50 years and older from the Household, Income and Labour Dynamics in Australia Survey (*n* = 5,500 individuals), where a question on sexual identity was asked twice in the study.

**Results:**

One percent of our sample reported a change in their sexual identity, which we grouped with individuals who reported as bisexual. Our sample comprised of 45.3% heterosexual men, 52.2% heterosexual women, 0.6% gay men, 0.6% lesbian women, 0.6% bisexual-plus men, and 0.6% bisexual-plus women. We found bisexual-plus men were vulnerable to loneliness as they aged. This group had the highest levels of loneliness at age 50, and differences compared with heterosexual men persisted over time. Loneliness of bisexual-plus men increased steeply from age 70. Socioeconomic and health statuses did not explain the increased loneliness of older bisexual-plus men. Lower social support and connectedness partly accounted for these disparities.

**Discussion and Implications:**

Findings are discussed with regards to existing research and theories on social disadvantage and resilience over the life course. We expand knowledge on factors explaining loneliness and how it varies in women and men by sexual identity.

Loneliness has been raised as a pressing issue among older adults (Blazer, 2020; van Tilburg et al., 2021; Wu, 2020). Identifying groups of older adults who are at a heightened risk of loneliness, as well as the mechanisms giving rise to these disparities, are therefore important endeavors. Such knowledge may inform targeted interventions to ameliorate loneliness and its pernicious effects. Existing research shows that loneliness varies across a range of individual characteristics, including sexual identity. Studies conducted in Western cultures have shown that older lesbian, gay, and bisexual (LGB) people are at a higher risk of loneliness in comparison to their heterosexual peers (Eres et al., 2021; Hsieh & Liu, 2021). The main reasons suggested for this disparity are lifetime exposure to structural stigma and sexual minority stressors (Fokemma & Kuyper, 2009; Kuyper & Fokkema, 2010). These could act to elevate loneliness among LGB people directly, as well as via disruptions to their social support networks, reduced socioeconomic opportunities, and impaired physical and mental health.

While scholars are showing increasing interest in the experiences of LGB people as they age, loneliness remains an understudied topic in this cohort. Drawing from the Iridescent Life Course perspective (Fredriksen-Goldsen et al., 2019) and minority stress theory (Meyer, 2003), we extend existing cross-sectional research and test whether the loneliness trajectories of older LGB people differ to those of their heterosexual counterparts. Drawing on a rich panel data set, we make three important contributions to the literature. First, we investigate potential differences in loneliness in later life at the intersection of sexual identity and gender—a core tenet of the Iridescent Life Course (Fredriksen-Goldsen et al., 2019). Second, we look beyond a single moment in time and examine loneliness longitudinally from age 50. Third, while previous research has speculated on the mechanisms driving sexual identity differences in loneliness, we empirically test whether socioeconomic factors, health statuses, and/or social connectedness and support explain variation in loneliness by sexual identity and gender in later life.

## The Iridescent Life Course and Minority Stress

To inform our investigation of loneliness among older LGB people, we draw on the Iridescent Life Course perspective (Fredriksen-Goldsen et al., 2019). Developed to guide research on the lives of gender and sexual minorities, the Iridescent Life Course framework emphasizes the intersectionality of gender and sexuality and the application of life course principles (Fredriksen-Goldsen et al., 2020). For example, the life course principle of “historical time” draws attention to the structural stigma and discrimination many LGB older adults have endured across their lives (e.g., job discrimination and marriage inequality). The life course principle of “the past affects the present” suggests that these constraints could have placed LGB people on a different trajectory as compared with their heterosexual counterparts and thus continued to shape their circumstances in later life, even when the sociocultural context may have since shifted toward greater inclusivity.

One life domain that is especially salient to loneliness is that of family and relationships. The life course principle of “linked lives” emphasizes the key role played by social networks and relationships in shaping life outcomes. On the one hand, it is often through close relationships and interpersonal interactions that the negative effects of a hostile sociocultural context are most acutely felt. For example, LGB people report less support from their parents and are more likely to be bullied or harassed by their peers compared to heterosexual people (Goodenow et al., 2016; Hsieh & Liu, 2021; Perales & Campbell, 2020). Thus, one potential mechanism for the production of higher loneliness among LGB older adults is lower social support. Yet on the other hand, embeddedness in supportive social networks such as LGB communities and “families of choice” can partially buffer the effects of a hostile sociocultural context and might engender resilience in some LGB people (Kuyper & Fokkema, 2010).

The minority stress model also emphasizes the potentially deleterious impacts of exposure to minority stressors on LGB people’s physical and mental health (Meyer, 2003). Several studies have documented the detrimental effects of minority stressors on the mental and physical well-being of LGB people (Flentje et al., 2020; Pitoňák, 2017). In turn, poorer physical and mental health are established risk factors for loneliness among older people (Luo et al., 2012). Discrimination and prejudice could also conceivably impede lifetime socioeconomic attainment among some LGB people—another established risk factor for loneliness later in life (Hansen & Slagsvold, 2016)—although evidence on this is mixed (e.g., Badgett, 2018). To our knowledge, only one study has examined the roles of health and socioeconomic factors in elevated loneliness among LGB people (Fokkema & Kuyper, 2009). This study found that health and socioeconomic status could not explain higher levels of loneliness in older LGB adults compared to their heterosexual peers. However, this study relied on a relatively small sample of LGB people and was unable to differentiate between different sexual identity and gender groups (e.g., bisexual women, gay men). Thus, further research on this issue is required.

## Bisexual Vulnerability

The broader LGB umbrella contains groups of individuals whose experiences may differ in meaningful ways. In our paper, we empirically test whether this is the case for loneliness. For example, across sexual minority groups, bisexual individuals may experience specific minority stressors related to their identity, including rejection and erasure from both heterosexual and lesbian/gay people (Mereish et al., 2017). Negative stereotypes frame bisexual individuals as undesirable partners to be in a relationship with and create barriers to finding acceptance and support within sexual minority communities (Dyar et al., 2018; Van Doan et al., 2019). This can lead to identity concealment and internalized stigma among bisexual people (Mereish et al., 2017), who report higher levels of identity confusion, are less likely to be out (i.e., publicly revealing their sexual identity), and report less connection to sexual minority communities than lesbian and gay people (Balsam & Mohr, 2007). Internalized stigma surrounding bisexual identity increases when individuals experience more frequent binegativity from others (Dyar et al., 2018).

Exposure to bisexual-specific minority stressors predicts poorer health over and above exposure to general sexual minority stressors (Katz-Wise et al., 2017), and there is consistent evidence that bisexual individuals experience poorer mental and physical health than both heterosexual and gay/lesbian individuals (Fredriksen-Goldsen et al., 2017; Mereish et al., 2017; Van Doan et al., 2019). Bisexual men and women are also significantly more likely to be socioeconomically disadvantaged than their lesbian, gay, or heterosexual peers (Badgett, 2018). Altogether, this evidence points toward an enhanced vulnerability to loneliness among bisexual people, and the need to consider their outcomes separately to those of gay and lesbian people—something that recent studies on loneliness among LGB older people were unable to do (Beam & Collins, 2019; Hsieh & Liu, 2021).

## The Intersections of Sexual Identity, Gender, and Race/Ethnicity

Beyond examining variation in experiences for LGB older adults, there is also some evidence to suggest that the experiences of LGB people differ by gender, although findings have been equivocal. It has been argued, for example, that bisexual women face less acceptance within sexual minority communities than bisexual men because historic lesbian separatist community politics may have heightened negative attitudes toward female bisexuality among lesbians, without a comparable counterpart of division among gay and bisexual men (Eliason, 2000; Salway et al., 2018). Consistent with this, Katz-Wise et al. (2017) found that bisexual women reported experiencing more antibisexual prejudice than bisexual men. Yet other (albeit older) studies have found the opposite, with greater prejudice toward bisexual men than bisexual women, particularly from heterosexual and gay male participants (Eliason, 2000; Mohr & Rochlen, 1999). In a recent study, Fredriksen-Goldsen et al. (2020) found evidence for somewhat greater vulnerability in bisexual men than women, with the men reporting higher rates of victimization, more identity stigma, and less identity affirmation and “outness.”

Gender also interacts with sexual orientation to shape the relative outcomes of lesbian women and gay men within socioeconomic, health, and relationship domains. For example, marginalization from hegemonic masculine norms appears to improve the academic performance of gay boys and men relative to their heterosexual male peers (Mittleman, 2022). In the labor market, meanwhile, marriage and motherhood result in a wage penalty for heterosexual but not lesbian women (Mize, 2016). In the family domain, gay men experience the greatest barriers to achieving parenthood, both in terms of pragmatics and stigma (Perales et al., 2020). Further, gay men are substantially less likely to be married/cohabiting than heterosexual men, while lesbian women marry/cohabit at similar rates to heterosexual women (Carpenter & Gates, 2008; Hseih & Ruther, 2016). Altogether, the interaction of sexual identity and gender seems to produce variable patterns of strengths and vulnerabilities in gay men and lesbian women. Whether this translates into differences in loneliness later in life remains to be seen.

Cultural and racial/ethnic background are other aspects of social identity that could plausibly intersect with sexual identity to shape levels of loneliness later in life. Existing evidence from samples of LGB adults, however, suggests no differences in loneliness along race/ethnicity lines. In their analyses of a national sample of almost 2,500 LGB older adults, Kim and Fredriksen-Goldsen (2016) found that non-Hispanic White people were more likely to be living with a partner or spouse than members of the other racial/ethnic groups. However, no significant differences in loneliness by race/ethnicity were found in their multivariate model. Likewise, Kertzner et al. (2009) found no race/ethnicity differences in social well-being (e.g., self-perceived social integration, acceptance) within their LGB sample of 396 LGB adults aged under 60 years.

## Hypotheses

Altogether, a synthesis of the above evidence led us to make the following two hypotheses:

**H1**: LGB older adults will report higher levels of loneliness than heterosexual older adults, with the greatest disparities evident in bisexual men and women

**H2**: Differences in loneliness between LGB and heterosexual older adults will be partially explained by differences in socioeconomic factors, health statues, and social support and connectedness

## Method

### Data

To test our hypotheses, we used data from Waves 1–19 of the Household Income and Labour Dynamics of Australia (HILDA) Survey. The HILDA Survey is a household panel study that has followed the same respondents since 2001 (for details, see Watson & Wooden, 2012, 2021). Data are collected annually from household members aged 15 years and older via face-to-face interviews and self-completed questionnaires. The survey covers topics within the domains of economic and personal well-being, labor market dynamics, and family life.

#### Sample

The original sample for the HILDA Survey comprised approximately 14,000 individuals from just over 7,500 households. Households were recruited via a complex, probabilistic sampling design, resulting in a sample that were largely representative of the Australian population aged 15 and older in 2001. Since that time, new respondents have joined the panel if they moved into a household where an existing respondent lived or if they lived in such a household and turned 15 years of age. In addition, a top-up sample of 2,000 responding households was added in 2011 (Watson, 2011).

For this study, we restricted our analytic sample to person-year observations from respondents aged 50 years and older at the time of data collection. This resulted in an initial analytic sample of 117,671 observations from 13,448 individuals. Given our focus on sexual identity, our sample was restricted to those older people who had reported their sexual identity at least once across the two waves in which the sexual identity question has so far been asked (Wave 12 in 2012 and Wave 16 in 2016). There were 4,674 individuals from our initial analytic sample who did not participate in either Waves 12 or 16, and therefore never reported their sexual identity. These individuals were dropped from our sample. A further 1,613 did participate in Waves 12 and 16 but did not provide data on their sexual identity—mostly due to failure to return the self-complete questionnaire (nonresponse to the self-completed questionnaire is 11%–12% in most waves of the HILDA Survey: see Watson & Wooden, 2015). Dropping these individuals further reduced our sample to 83,563 observations from 7,161 people. Person-year observations with missing data on the outcome variable (loneliness) or other explanatory variables (health, socioeconomic status, social connectedness/support) were also excluded, resulting in a final analytic sample of 77,775 observations from 7,123 individuals aged between 50 and 100 years (mean = 62.9). The number of observations contributed by each member of our sample ranged from 1 to 19, with a mean of 10.9. Descriptive statistics for our analytic sample can be found in Table 1.

**Table 1.** Sample Descriptive Statistics

Variable	%	Mean (*SD*)
Loneliness		2.5 (1.8)
Sexual identity + gender		
Heterosexual man	45.3	
Heterosexual woman	52.2	
Bisexual-plus man	0.6	
Bisexual-plus woman	0.7	
Gay man	0.6	
Lesbian woman	0.6	
Age		62.9 (9.5)
Annual personal income ($10,000’s)		4.1 (4.2)
Highest educational qualification		
Postgraduate degree (masters or doctorate)	5.2	
Graduate diploma/certificate	7.0	
Bachelor’s degree or honors	10.8	
Advanced diploma, diploma (nonuniversity)	10.9	
Certificate III or IV (nonuniversity)	21.9	
High school (Year 12)	8.1	
Less than Year 12	36.1	
Physical functioning (0–100)		75.8 (24.3)
Mental health (0–100)		76.6 (16.9)
Social connectedness (0–7)		4.0 (1.5)
Social support (1–7)		5.5 (1.0)
Observations	77,775	
Individuals	7,123	

*Notes*: *SD* = standard deviation.

Source: Household, Income and Labour Dynamics in Australia Survey, Waves 1 (2001) to 19 (2019).

### Measures

#### Loneliness

Our dependent variable was loneliness. Respondents were asked how much they agreed or disagreed with the following statement: “I often feel very lonely.” Responses were measured on a Likert-type scale ranging from 1 (*strongly disagree*) to 7 (*strongly agree*), with higher scores reflecting greater loneliness. The distribution of responses to this item was highly skewed, with individuals scoring 1 or 2 in approximately 64% of observations. Thus, to reduce skew, we used a log transformation of the outcome variable in our analyses.

#### Sexual identity

To measure sexual identity, respondents were asked “Which of the following categories best describes how you think of yourself?” Possible responses included “Heterosexual or Straight,” “Gay or Lesbian,” “Bisexual,” “Other,” “Unsure/don’t know,” and “Prefer not to say.” Approximately 13% of older people did not answer the sexual identity question in each wave, primarily because they did not respond to the self-completed questionnaire containing the item. Among those people aged 50 and older who did respond to the question, around 1% selected “other,” 1% were unsure, and 3.6% preferred not to answer. Given the impossibility of interpreting the meaning of these responses, they were recoded as missing. As sexual identity was asked twice, respondents only needed to have a valid response to the question in one of the two waves to be included in our sample.

Of those respondents who provided a valid response in both waves, the vast majority reported the same sexual identity in both instances. However, around 1% of our analytic sample did not. Specifically, 0.5% changed their response from heterosexual to bisexual and 0.5% changed from bisexual to heterosexual. There was only once instance of a respondent changing from bisexual to lesbian/gay, and no instances of change between lesbian/gay and heterosexual in either direction. Unfortunately, our data do not offer any insights into why some respondents changed between heterosexual and bisexual identities. While some may have experienced a genuine change in their sexual attractions or behavior, others may have changed their response because they felt more comfortable “coming out” and reporting a bisexual identity over time. Given that all respondents who changed their sexual identity reported a bisexual identity in one of the two waves, we decided to combine the individuals who changed their identity with the bisexual individuals in a “Bisexual-plus (Bi+)” group. While we would have preferred to separate these groups, small numbers made this unfeasible.

We further categorized respondents according to their gender (man/woman) as reported in the household grid, as more nuanced data on gender identity are not currently collected in the HILDA Survey. This resulted in six groups for our analysis: lesbian women (*n* = 40, 0.6%), gay men (*n* = 46, 0.6%), bisexual-plus women (*n* = 53, 0.7%), bisexual-plus men (*n* = 43, 0.6%), heterosexual women (*n* = 3,717, 52.2%), and heterosexual men (*n* = 3,224, 45.3%).

#### Health

To measure physical health, we used respondents’ scores on the Short Form Health Survey physical functioning scale (Ware, 2000). Respondents were asked to rate the extent to which their health limits their performance of 10 everyday activities, such as lifting or carrying groceries, climbing one flight of stairs, and walking more than 1 km. Scores were calculated and transformed according to the manual, to create a final scale ranging from 0 (*worst physical functioning*) to 100 (*best physical functioning*). To measure mental health, we used respondents’ scores on the Short Form Health Survey mental health scale (Ware, 2000), which comprises five questions asking how nervous happy, down, and calm and peaceful a person had been feeling over the past 4 weeks. Scores were calculated and transformed according to the manual, to create a final scale ranging from 0 (*worst mental health*) to 100 (*best mental health*).

#### Socioeconomic status

Socioeconomic status was measured using annual personal income (in the $10,000’s) and highest educational qualification, classified as: (a) masters or doctorate degree; (b) graduate diploma/certificate; (c) bachelor’s degree or honors; (d) advanced diploma, diploma (nonuniversity); (e) certificate III or IV (nonuniversity); (f) high school (Year 12); and (g) less than Year 12.

#### Social connectedness and support

To measure social connectedness, we created a count variable ranging from 0 to 7. We utilized items in the data set that concorded with those used in prior studies (Cornwell et al., 2008; Fokemma & Kuyper, 2009; Hodge et al., 2013). Respondents scored one point for each of the following: (a) is married or cohabiting, (b) does not live alone, (c) has contact with friends or relatives from other households at least once a week, (d) is employed, (e) volunteers, (f) is a member of a club/community group, and (g) is satisfied with feeling part of the local community. Level of social support was measured using the mean score from nine items, for example, “I seem to have a lot of friends,” “There is someone who can always cheer me up when I’m down,” and “I have no one to lean on in times of trouble” (reversed). Responses were measured on a Likert-type scale of 1 (*strongly disagree*) to 7 (*strongly agree*). Items were reversed as appropriate, and mean scores were calculated for each respondent. Higher scores on the index indicated higher levels of perceived social support. There was a weak, positive correlation between our measures of social connectedness and social support (*r* = 0.27, *p* < .001).

### Statistical Analyses

To test our hypotheses, we estimated a series of five growth models within the multilevel framework. All analyses were conducted using the *mixed* command in Stata 16. Age in years was our Level-1 variable capturing time. To facilitate interpretation of the intercept, we centered age on 50 years. In our first model, we tested whether intercepts and/or slopes of loneliness trajectories differed between older Australians according to their sexual identity group. Linear and quadratic terms for age were found to be significant and were thus retained, whereas cubic terms were not. We then added each block of predictor variables (i.e., health, socioeconomic status, and social connectedness/support) separately, to assess if associations between sexual identity and loneliness were attenuated, and if so, to what extent. In the final model we included the three blocks of predictors simultaneously, to see which remained significant when all other variables were accounted for.

Heterosexual men were the reference group in all our models. Model intercepts therefore represent the predicted log of loneliness for heterosexual men at age 50, while the intercept coefficients for the other sexual identity groups show the differences in their predicted log of loneliness at age 50 compared to heterosexual men. Likewise, the model rates of change (linear and quadratic terms) represent the loneliness curve for heterosexual men from the age of 50. Meanwhile, the rate of change coefficients for the other sexual identity groups shows the differences in their loneliness growth curves compared to that of heterosexual men.

## Results

### Bivariate Associations

We began by estimating bivariate associations between sexual identity and loneliness, health status, socioeconomic status, and social connectedness/support variables. Full results are presented in Supplementary Table 1. To examine differences between the sexual identity groups at the bivariate level we conducted omnibus tests (analysis of variance for continuous variables and chi-square for categorical variables), followed up when significant with post hoc pairwise comparisons. We found diverging and complex patterns of strengths and vulnerabilities across the different sexual identity groups. *Heterosexual men* were advantaged on many (but not all) measures. They had the lowest levels of loneliness, the highest annual income, the highest social connectedness, and the best mental health. They reported higher levels of social support than bisexual-plus men but lower levels than heterosexual and lesbian women. *Bisexual-plus men* reported the highest levels of loneliness and the lowest levels of social support. They reported lower social connectedness than all other groups except gay men. *Bisexual-plus women* had the lowest personal income and reported lower social connectedness and support than heterosexual and lesbian women. *Lesbian women* had the poorest physical and mental health on average. Yet at the same time, they were more likely than any other group to hold a university qualification, and their average personal annual income was second only to heterosexual men. Along with heterosexual women, they also reported the highest level of social support of all the sexual identity groups. *Heterosexual women* were the least likely to have completed high school and had the second lowest income. Yet they had the highest levels of social support (equal to lesbian women), and the second highest level of social connectedness.

### Loneliness Trajectories

**Figure 1.**
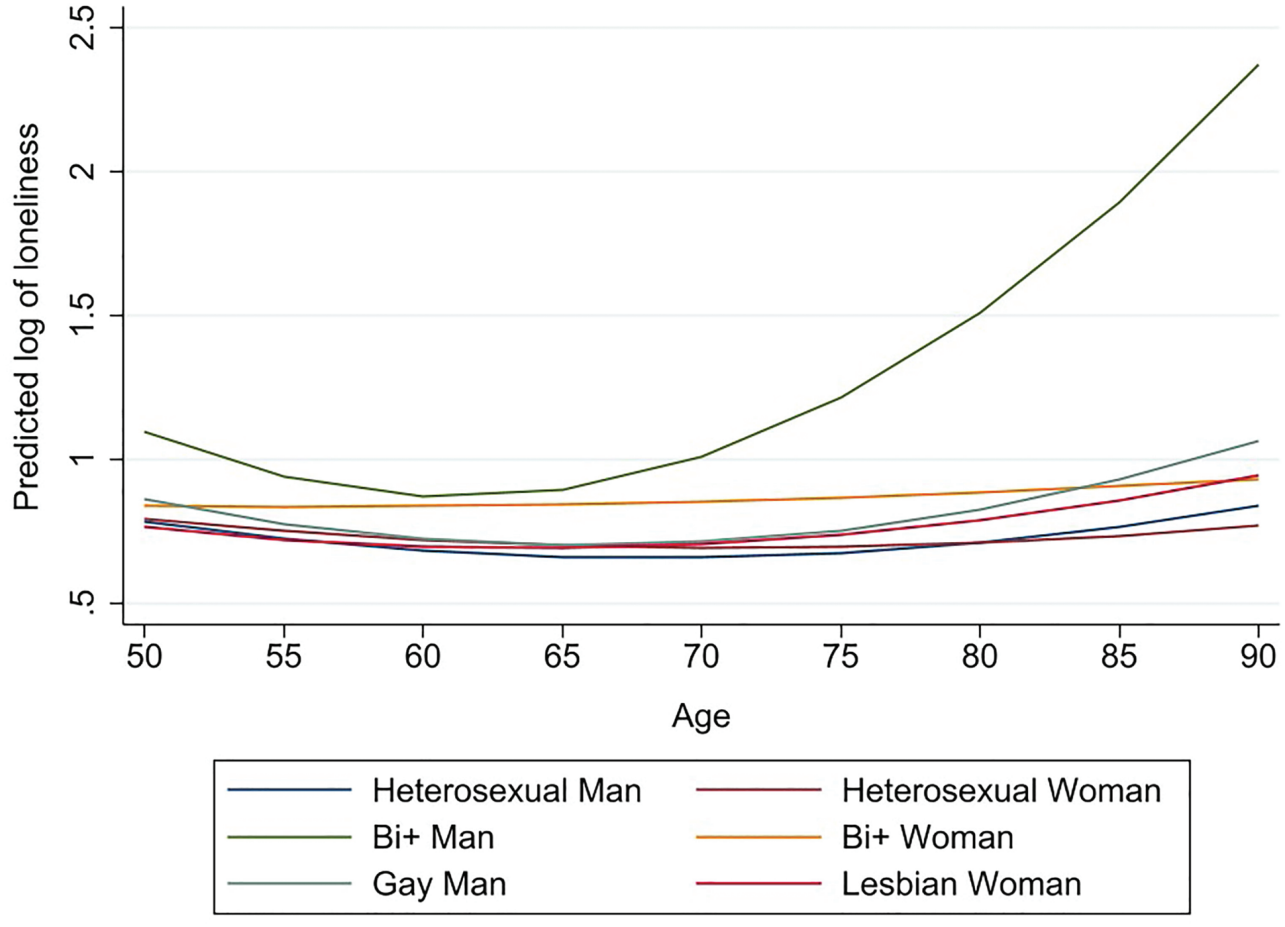
Growth trajectories of loneliness conditioned on sexual identity for older men and women (from Model 1).

Estimates from our five growth models of loneliness are presented in Table 2. The results from Model 1 represent the predicted loneliness trajectories of Australians from the age of 50 conditioned on their sexual identity and gender. These trajectories are displayed graphically in Figure 1. At age 50, the starting point for our growth curves (i.e., the intercept), only bisexual-plus men had significantly higher levels of loneliness than heterosexual men (our reference group). The rate of change in loneliness for older bisexual-plus men also differed significantly to that for older heterosexual men. The trajectory of bisexual-plus men was characterized by a subtle decline between ages 50 and 65, followed by a steep incline thereafter. In contrast, the loneliness trajectories of the other sexual identity groups were generally flat. Thus, our first hypothesis was only partially supported, with bisexual-plus men, but none of the other sexual minority groups, reporting significantly higher levels of loneliness than heterosexual men.

**Table 2.** Growth Model of Loneliness Conditioned on Sexual Identity Group

Model variable	Model 1		Model 2		Model 3		Model 4		Model 5	
	Est.	*SE*	Est.	*SE*	Est.	*SE*	Est.	*SE*	Est.	*SE*
Intercept	0.783***	0.013	0.843***	0.016	1.724***	0.018	2.340***	0.017	2.755***	0.020
Sexual identity (ref = hetero. man)										
Heterosexual woman	0.008	0.017	−0.000	0.017	−0.018	0.015	0.086***	0.013	0.057***	0.013
Bisexual-plus man	0.313**	0.108	0.304**	0.108	0.330***	0.094	0.250**	0.084	0.264***	0.079
Bisexual-plus woman	0.053	0.099	0.059	0.099	0.017	0.087	0.016	0.078	−0.006	0.073
Gay man	0.076	0.104	0.084	0.103	0.035	0.090	0.093	0.081	0.072	0.076
Lesbian woman	−0.019	0.104	−0.005	0.103	−0.081	0.090	0.076	0.080	0.029	0.075
Rate of change (age)—linear	−0.014***	0.001	−0.014***	0.001	−0.009***	0.001	−0.009***	0.001	−0.007***	0.001
Sexual identity (ref = hetero. man)										
Heterosexual woman	0.005*	0.002	0.004*	0.002	0.004*	0.002	0.002	0.002	0.002	0.002
Bisexual-plus man	−0.027*	0.012	−0.026*	0.012	−0.037***	0.011	−0.039***	0.010	−0.043***	0.010
Bisexual-plus woman	0.013	0.011	0.012	0.011	0.010	0.010	0.012	0.010	0.011	0.009
Gay man	−0.006	0.014	−0.006	0.014	−0.000	0.013	−0.011	0.012	−0.007	0.012
Lesbian woman	0.003	0.013	0.004	0.013	−0.001	0.012	0.001	0.012	−0.001	0.011
Rate of change (age)—quadratic	0.000***	0.000	0.000***	0.000	0.000***	0.000	0.000***	0.000	0.000***	0.000
Sexual identity (ref = hetero. man)										
Heterosexual woman	−0.000**	0.000	−0.000**	0.000	−0.000*	0.000	−0.000*	0.000	−0.000	0.000
Bisexual-plus man	0.001***	0.000	0.001***	0.000	0.002***	0.000	0.002***	0.000	0.002***	0.000
Bisexual-plus woman	−0.000	0.000	−0.000	0.000	−0.000	0.000	−0.000	0.000	−0.000	0.000
Gay man	0.000	0.001	0.000	0.001	0.000	0.000	0.000	0.000	0.000	0.000
Lesbian woman	−0.000	0.001	−0.000	0.001	0.000	0.001	−0.000	0.000	−0.000	0.000
Socioeconomic status										
Personal annual income ($10,000’s)			−0.003***	0.001					−0.000	0.001
Highest educational qualification (ref = less than Year 12)										
Masters or doctorate			−0.092***	0.025					−0.017	0.018
Graduate diploma/certificate			−0.111***	0.023					−0.027	0.016
Bachelor’s degree or honors			−0.090***	0.020					−0.019	0.013
Advanced diploma, diploma			−0.078***	0.019					−0.021	0.013
Certificate III or IV			−0.035*	0.015					−0.016	0.010
High school (Year 12)			−0.029	0.022					−0.003	0.015
Health status										
Physical functioning (0–100)					−0.001***	0.000			−0.000	0.000
Mental health (0–100)					−0.012***	0.000			−0.008***	0.000
Social support and connectedness										
Social connectedness (0–7)							−0.045***	0.002	−0.033***	0.002
Social support index (1–7)							−0.264***	0.003	−0.230***	0.003
Model fit statistics										
AIC	123,580.3		123,530.8		118,561.7		113,446.2		110,747.2	
BIC	123,784.0		123,808.7		118,784.0		113,668.5		111,062.1	

*Notes*: *SE* = standard error.

Statistical significance: *p < .05. **p < .01. ***p < .001.

Source: Household, Income and Labour Dynamics in Australia Survey, Waves 1 (2001) to 19 (2019). 77,775 person-year observations.

In Model 2, we added the socioeconomic variables. Both income and educational attainment were significant, negative correlates of loneliness, with higher income and education associated with lower levels of loneliness. However, adding these two variables did little to improve model fit, as indicated by changes to the Akaike information criterion and Bayesian information criterion shown at the bottom of Table 2. Nor did it attenuate differences in the underlying trajectories of bisexual-plus compared to heterosexual men. In Model 3 we added the heath variables instead of the socioeconomic variables. As expected, relationships between health and loneliness were significant, with poorer physical and mental health associated with greater loneliness. While Model 3 better fit the data than Models 1 or 2, the health variables also failed to account for differences in the underlying loneliness trajectories of bisexual-plus versus heterosexual men. In Model 4, we added the social support and connectedness variables. Loneliness decreased as levels of social connectedness and perceived social support increased. The addition of the social connectedness and support variables in Model 4 improved model fit more than the addition of the socioeconomic or health variables had in Models 2 and 3. Further, in partial support of our second hypothesis, social connectedness and support partly attenuated differences in the loneliness trajectories of bisexual-plus compared to heterosexual men. For example, the difference in the intercept for bisexual-plus men decreased from 0.31 in Model 1 to 0.26 in Model 4 when the social connectedness/support variables were added. However, the differences between these two groups remained significantly different to zero.

In Model 5, we added the socioeconomic, health, and social connectedness/support variables simultaneously. The Akaike information criterion and Bayesian information criterion indicated that this model was a slight improvement over Model 4, making it the model that best fit our data. In this final model, only mental health, social connectedness, and social support remained significant predictors of loneliness. Associations between socioeconomic status and loneliness were largely attenuated and no longer significantly different to zero. In contrast, associations between sexual identity and loneliness remained significant. This indicates that the loneliness disparities we observe between bisexual-plus and heterosexual men are largely the product of factors unaccounted for in our models.

## Discussion

Our study has contributed to existing literature on variation in outcomes by sexual identity among older adults. Drawing on a rich panel data set with a sexual identity question and repeated measures of loneliness, we were able to empirically test whether loneliness trajectories differ by sexual identity between the ages of 50 and 100 years. Contrary to our expectations, we found that older gay men, lesbian women, and bisexual-plus women do not experience higher levels of loneliness than heterosexual men and women. Given the homophobic social context in which these older people lived much of their lives—a world in which marriage equality was still a far-distant dream and gay hates crimes occurred unchecked—this finding is somewhat surprising. It arguably reflects the resilience many LGB people develop across their lives through connections with community and the creation of “families of choice.” In support of this, our bivariate analyses showed no differences between lesbian and heterosexual women, or between gay and heterosexual men, in average levels of social support. On other measures, such as the attainment of university qualifications, gay and lesbian older adults outperformed their heterosexual counterparts. These results provide an important counternarrative to the deficit discourse that often dominates research on LGB people.

We did find one exception to the above: bisexual-plus men, who reported significantly higher levels of loneliness than heterosexual men. While bisexual-plus men emerged as a particularly vulnerable group with regards to loneliness, health, and socioeconomic status did little to explain this. This was again contrary to our expectations, although it concords with the findings of an earlier Dutch study (Fokkema & Kuyper, 2009). We did, however, find that bisexual-plus men were disadvantaged compared to others with regards to social connectedness and support, and that this partly explained their disparities in loneliness. This would seem consistent with prior research showing that bisexual people are subjected to negative stereotypes and “double discrimination” from both heterosexual and lesbian/gay communities (Mereish et al., 2017). A previous study also found that older bisexual men report fewer social resources and more exposure to minority stressors than bisexual women (Fredriksen-Goldsen et al., 2020). Taken together, this indicates the urgent need for further research into the experiences of bisexual men as they age, and the implementation of effective strategies to increase their social connectedness and support.

A strength of our paper was the fact that we considered the outcomes of bisexual adults separately from gay and lesbian adults, something that recent papers on this topic were unable to do (Beam & Collins, 2019; Hsieh & Liu, 2021). In addition, we were able to identify older adults who changed their sexual identity across survey waves. While only comprising a small proportion of our sample (1%), the presence of these individuals reinforces another key tenet of the Iridescent Life Course—that of fluidity. While changes in sexual identity are most often observed among younger cohorts (Campbell et al., 2021), our study shows that it also occurs in a small proportion of older adults. Unfortunately, small numbers meant we were unable to analyze these individuals as a separate group. Future research should investigate the experiences of this small but potentially vulnerable group of older adults to understand what these changes in identity mean for them. It may be the case that some people experience changes in their sexual attractions or behaviors later in life, while others might just feel more comfortable disclosing their nonheterosexual identity with time. Altogether, our study reinforces the necessity of collecting repeated measurements of sexual identity, separating bisexual from lesbian and gay identities in analyses, and considering the possibility of changes in identity over time even within samples of older adults.

While we have advanced existing knowledge on the outcomes of LGB people as they age, our study is not without some limitations. First, the data we used did not contain measures of internalized stigma, identity concealment, or exposure to other minority stressors such as discrimination. This precluded us from testing all potentially important pathways linking gender and sexual identity to loneliness. Further research is needed to address this. Second, accounting for intersections between biographical and historical time is a key tenet of the Iridescent Life Course. As societal attitudes toward sexual minorities shift, there may be differences in the experiences of LGB older adults across successive generations. Unfortunately, we could not differentiate participants by birth cohort as cell sizes would have become too small. Future research that draws on a larger sample could test for such differences. Third, we were unable to examine the experiences of transgender or gender diverse individuals as the HILDA Survey has not collected these data to date. Further studies that draw on data sets containing these measures are sorely needed.

Notwithstanding these limitations, the findings of our study have important ramifications. The bisexual-plus older men in our sample reported levels of loneliness that were not only higher than all other groups, but that also escalated markedly from age 70 onwards. As we continue to learn more about the experiences of older gender and sexual minorities, this knowledge must be translated into policy and practice. Based on the findings we have reported here, this means identifying strategies for increasing both the acceptance of bisexuality in the general population, and the social support and connectedness available to bisexual men as they age.

## Supplementary Material

gnac058_suppl_Supplementary_Material
