# Supplementary material for: Trajectories of Loneliness Among Older Women and Men: Variation by Sexual Identity?
Source: Gerontologist. 2022 Apr 22;63(2):328–37. doi: 10.1093/geront/gnac058 (PMC9960021; doi:10.1093/geront/gnac058)
Supplement: gnac058_suppl_Supplementary_Material [file gnac058_suppl_supplementary_material.docx]

**Online Supplementary Material**

Supplementary Table 1. Bivariate associations between sex/sexual identity and model variables

| Variable | Heterosexual Men  (a) | Heterosexual Women  (b) | Bi+ Men  (c) | Bi+ Women  (d) | Gay Men  (e) | Lesbian Women  (f) |
| --- | --- | --- | --- | --- | --- | --- |
|  | Mean (SD) | Mean (SD) | Mean (SD) | Mean (SD) | Mean (SD) | Mean (SD) |
| Loneliness (1-7) | 2.5 (1.7)^bcd^ | 2.6 (1.8)^acd^ | 3.2 (1.9)^abdef^ | 2.8 (1.8)^abc^ | 2.7 (1.9)^c^ | 2.7 (2.0)^c^ |
| Age | 62.7 (9.3)^bef^ | 63.1 (9.6)^aef^ | 62.7 (9.0)^ef^ | 62.8 (9.5)^ef^ | 60.3 (7.8)^abcd^ | 58.6 (7.6)^abcd^ |
| Annual personal income ($10,000’s) | 4.9 (5.1)^bcd^ | 3.3 (3.2)^acef^ | 4.4 (3.2)^abd^ | 2.9 (2.3)^acef^ | 4.4 (3.0)^bd^ | 4.8 (3.6)^bd^ |
| Physical functioning (0-100) | 78.7 (23.2)^bdf^ | 73.4 (24.9)^ace^ | 79.0 (21.9)^bdf^ | 74.3 (24.4)^ace^ | 79.0 (25.2)^bdf^ | 72.0 (27.8)^ace^ |
| Mental health (0-100) | 77.7 (16.4)^bcdf^ | 75.8 (17.2)^acf^ | 73.0 (19.2)^ab^ | 74.8 (16.4)^af^ | 75.5 (16.5)^f^ | 69.8 (17.5)^abde^ |
| Social connectedness (0-7) | 4.2 (1.4)^bcdef^ | 3.9 (1.5)^acde^ | 3.4 (1.7)^abef^ | 3.5 (1.4)^abe^ | 2.7 (1.4)^abcdf^ | 3.7 (1.6)^ace^ |
| Social support (1-7) | 5.3 (1.0)^bcf^ | 5.6 (1.0)^acde^ | 4.9 (1.0)^abdef^ | 5.3 (1.0)^bcf^ | 5.3 (1.1)^bcf^ | 5.6 (1.1)^acde^ |
|  |  |  |  |  |  |  |
| Highest educational qualification | % | % | % | % | % | % |
| Masters or doctorate | 6.4^bcf^ | 3.8^acdef^ | 18.9^abde^ | 6.2^bcf^ | 8.6^bcf^ | 15.7^abde^ |
| Graduate diploma/certificate | 6.5^bcef^ | 7.5^acef^ | 3.5^abdef^ | 6.2^cef^ | 10.2^abcd^ | 11.9^abcd^ |
| Bachelor’s degree or honours | 11.0^bcdf^ | 10.4^acdf^ | 5.3^abdef^ | 15.3^abcf^ | 11.6^cf^ | 24.9^abcde^ |
| Advanced diploma, diploma (non-university) | 11.9^bcdf^ | 10.1^a^ | 7.5^a^ | 8.2^a^ | 10.4 | 8.3^a^ |
| Certificate III or IV (non-university) | 30.2^bcdef^ | 14.8^acdef^ | 24.8^abf^ | 22.4^abf^ | 24.7^abf^ | 9.1^abcde^ |
| High school (Year 12) | 7.2^bcdef^ | 8.6^acde^ | 11.8^abe^ | 12.5^abe^ | 19.5^abcdf^ | 9.9^ae^ |
| Year 11 and below | 26.6^bef^ | 44.7^acdef^ | 28.3^bef^ | 29.3^bef^ | 15.0^abcd^ | 20.2^abcd^ |
| Observations | 35,168 | 40,745 | 509 | 550 | 441 | 362 |
| Individuals | 3,224 | 3,717 | 43 | 53 | 46 | 40 |

*Note*. Source: Household, Income and Labour Dynamics in Australia Survey, Waves 1(2001) to 19(2019). Omnibus tests (chi-squared for categorical variables and ANOVA tests for continuous variables) showed significant differences on all model variables by sexual identity. Post-hoc pairwise comparisons were conducted (two-sample proportions z-tests for categorical variables and Tukey comparisons of means for continuous variables). Superscript letters denote groups with significantly different proportions/means (*p* < .05).
